# Supplementary material for: Generalized reliability based on distances
Source: Biometrics. 2020 May 8;77(1):258–70. doi: 10.1111/biom.13287 (PMC7984087; doi:10.1111/biom.13287)
Supplement: Supplementary file 1 — Web Appendix A, referenced in Section 1, Web Appendix B, referenced in Section 4, Web Appendix C, referenced in Section 6.2, and Web Appendices D and E, referenced in Section 7, along with a brief guide to the R code, are available with this paper at the Biometrics website on Wiley Online Library. A package for R (R Core Team, 2019) implementing the methods of this paper is available at https://github.com/wtagr/dbicc. [file BIOM-77-258-s002.pdf]

Supporting Information for "Generalized reliability based on distances"

Meng Xu[1], Philip T. Reiss[1], and Ivor Cribben[2]

[1]Department of Statistics, University of Haifa, Haifa 31905, Israel
[2]Department of Accounting, Operations, and Information Systems, University of Alberta School of Business, Edmonton, AB T6G 2R6, Canada

A A heuristic measure of overall connectivity

For purposes of choosing high- and low-connectivity correlation matrices to be displayed in Fig. 1, we defined $-\log|\boldsymbol{R}|$ as a global connectivity score for a given correlation matrix $\boldsymbol{R}$. This score equals the LogDet divergence (Kulis et al., 2009) between $\boldsymbol{R}$ and the identity matrix, and its statistical properties have been investigated by Jiang (2019). As can be seen in Fig. 1, the score $-\log|\boldsymbol{R}|$ distinguishes clearly between fMRI scans evincing high versus low degrees of connectivity.

B The $\sqrt{1-r}$ distance

The $\sqrt{1-r}$ distance introduced in Section 4 follows Shehzad et al. (2014), who used $\sqrt{2(1-r)}$ as a distance among fMRI connectivity matrices (the multiplicative constant $\sqrt{2}$ clearly has no effect on dbICC). To explain the rationale for this, we consider a collection $\boldsymbol{R}_1, \ldots, \boldsymbol{R}_N$ of correlation matrices, and let r_{ij} denote the Pearson correlation between the lower-triangular elements of $\boldsymbol{R}_i$ and those of $\boldsymbol{R}_j$. Since the correlation-of-correlations matrix $\boldsymbol{C} = (r_{ij})_{1\le i,j\le N}$ is positive semidefinite, a result of Gower (1966) (see Theorem 14.2.2 of Mardia et al., 1979) implies that the distance matrix $\boldsymbol{D} = [\sqrt{2(1-r_{ij})}]_{1\le i,j\le N}$ is Euclidean, that is, there exist N points in a Euclidean space whose inter-point distances are given by $\boldsymbol{D}$. An alternative correlation-based distance would be $1-r$ (Walther et al., 2016), but we opted for $\sqrt{1-r}$ since this Euclidean property makes it a natural comparator for the Euclidean (ℓ_2) distance between (vectorized) correlation matrices.

C Further results on time series length and SNR

Fig. 6 presented log-log plots relating time series length to the SNR $\frac{\rho}{1-\rho}$, where ρ is based on the ℓ_2 distance. Figure W1 presents analogous plots for the ℓ_1 and $\sqrt{1-r}$

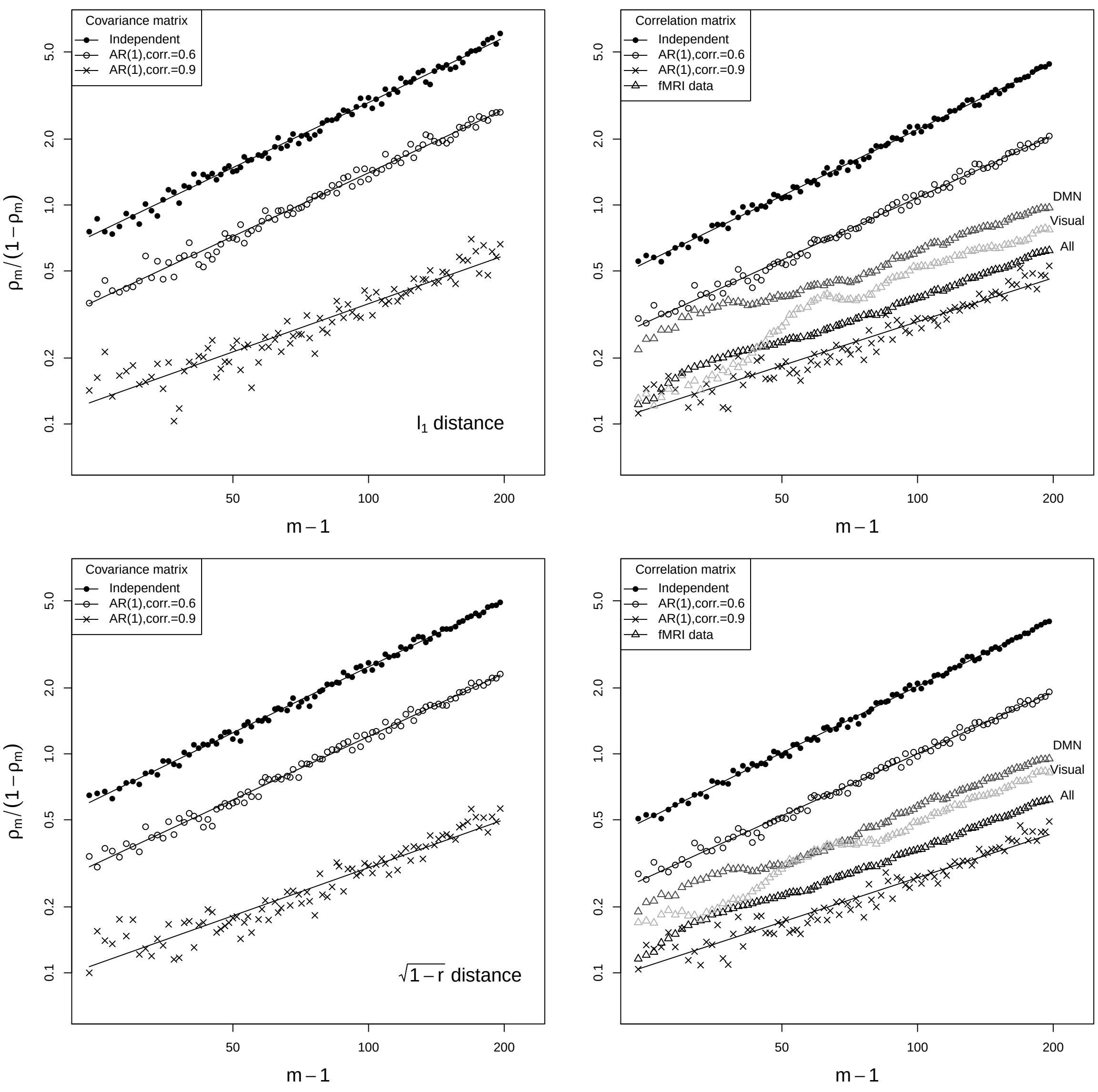

Figure W1: Log-log plots as in Fig. 6, but for the ℓ_1 and $\sqrt{1-r}$ distances.

distances introduced in Section 4. The intercepts and slopes of the best-fit lines in both figures are given in Table W1. In Section 6.2 we argued, in light of (17), that for covariance estimation with independent observations and the ℓ_2 distance, we should observe a slope near 1. In Table W1 we observe slopes near 1 with independent data using all three distances, and for correlation as well as covariance matrix estimation.

The fMRI results in Fig. W1 are similar to those in Fig. 6. While the visual network slopes in Table W1 are noticeably higher than for the other two sets of ROIs, this appears to be due primarily to the unstable small-m portion of the plots.

Distance	Setting (simulation/data)	Covariance Intercept (SE)	Covariance Slope (SE)	Correlation Intercept (SE)	Correlation Slope (SE)
ℓ_2	Independent	-3.435 (0.045)	0.997 (0.010)	-3.903 (0.029)	1.018 (0.007)
	VAR(1), $\phi = 0.6$	-4.136 (0.056)	0.986 (0.013)	-4.358 (0.037)	0.960 (0.008)
	VAR(1), $\phi = 0.9$	-4.397 (0.122)	0.736 (0.028)	-4.428 (0.086)	0.687 (0.020)
	fMRI data (all 333)			-4.283 (0.028)	0.716 (0.006)
	(DMN)			-3.627 (0.037)	0.676 (0.009)
	(visual)			-4.759 (0.072)	0.872 (0.017)
ℓ_1	Independent	-3.470 (0.043)	0.987 (0.010)	-3.840 (0.029)	1.006 (0.007)
	VAR(1), $\phi = 0.6$	-4.104 (0.053)	0.965 (0.012)	-4.283 (0.038)	0.947 (0.009)
	VAR(1), $\phi = 0.9$	-4.404 (0.117)	0.731 (0.027)	-4.293 (0.087)	0.666 (0.020)
	fMRI data (all 333)			-4.185 (0.029)	0.699 (0.007)
	(DMN)			-3.498 (0.038)	0.654 (0.009)
	(visual)			-4.864 (0.081)	0.897 (0.019)
$\sqrt{1-r}$	Independent	-3.690 (0.031)	1.000 (0.007)	-3.942 (0.025)	1.010 (0.006)
	VAR(1), $\phi = 0.6$	-4.240 (0.042)	0.960 (0.010)	-4.337 (0.035)	0.942 (0.008)
	VAR(1), $\phi = 0.9$	-4.556 (0.101)	0.729 (0.023)	-4.401 (0.083)	0.673 (0.019)
	fMRI data (all 333)			-4.367 (0.026)	0.733 (0.006)
	(DMN)			-4.054 (0.041)	0.757 (0.009)
	(visual)			-4.404 (0.046)	0.803 (0.011)

Table W1: Intercepts and slopes of the best fit lines in Figures 6 and W1.

D Spearman-Brown formula for curve estimation

Let $T_1, \dots, T_I$ be a random sample of curves drawn from $\mathcal{H} = L^2[0,1]$, and for $i \in \{1, \dots, I\}$, let $X_{i1}, \dots, X_{iJ_i}$ be penalized spline estimates of T_i based on m noisy observations. More specifically, for $r = 1, \dots, m$, let $u_r = \frac{r-1}{m-1}$ and $y_{ijr} = T_i(u_r) + \nu_{ijr}$, where the ν_{ijr} are independently sampled from the $N(0, \sigma^2)$ distribution for some $\sigma^2 > 0$. Then $X_{ij}(\cdot)$ is an estimate of $T_i(\cdot)$ by penalized spline smoothing of the points $(u_1, y_{ij1}), \dots, (u_m, y_{ijm})$. As $m \to \infty$, the optimal mean squared L^2 error in estimating T_i by cubic splines is $O(m^{-8/9})$, provided the number of knots $k = O(m^{1/9})$, although the effect of automatic smoothing parameter selection on this convergence rate is somewhat complex (Wood et al., 2016, Supplementary Appendices A and B). This suggests that $\Delta_\varepsilon(m) \propto m^{-8/9}$, and hence a linear model fit to the points $[\log(m), \log\{\hat{\rho}_m/(1-\hat{\rho}_m)\}]$ should have slope approximately $\frac{8}{9}$.

We sought to verify this by a simulation study with $I = 30$ and J_i fixed at a constant J of either 2 or 10. For $i = 1, \dots, 30$, we generated a random function $T_i(u) = a \cdot \sin(5u) + b \cdot \cos(2u)$, where a and b are drawn independently from the standard normal distribution. Then, for each i, J noisy realizations of T_i were generated with $\sigma^2 = 0.5$, and function estimates $X_{i1}, \dots, X_{i,J}$ were obtained by penalized splines based on 31 values of the number m of observations, ranging from 148 to 2981 and roughly uniformly spaced on the log scale. The fitted line in Fig. W2 for $J = 2$ has slope 0.894 with standard error 0.045, while that for $J = 10$ has slope 0.838 with standard error 0.043. Both slopes, then, are quite consistent with the theoretical value $\frac{8}{9}$.

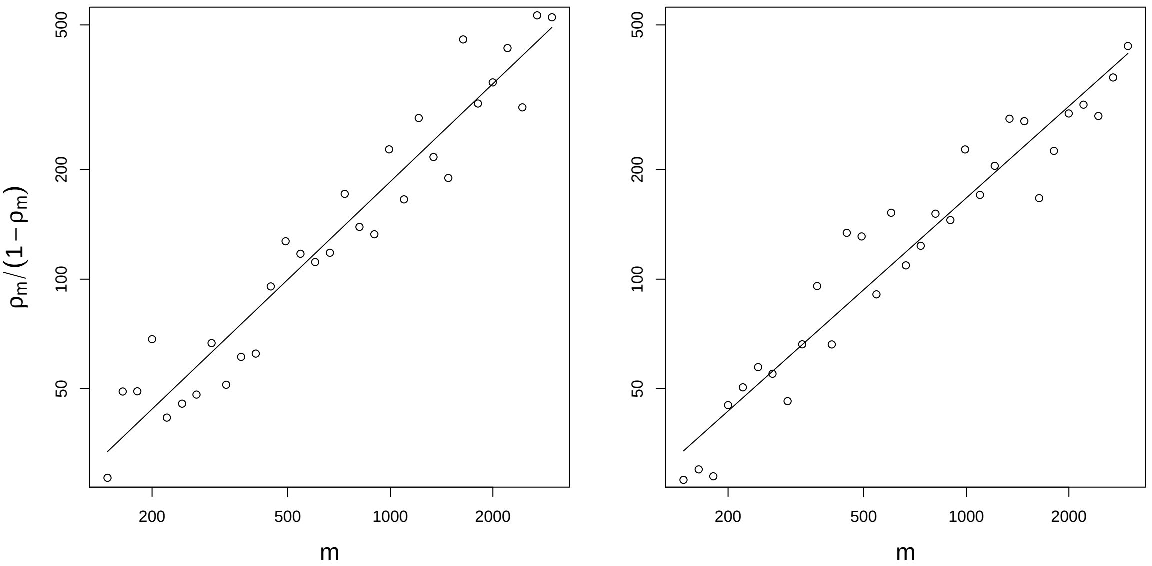

Figure W2: Measurement intensity versus SNR for the curve estimation simulation, with $J = 2$ (left) and $J = 10$ (right). Both axes are on the log scale, resulting in a linear relationship.

E Signature data

Next we consider part of the data from the First International Signature Verification Competition, available at `http://www.cse.ust.hk/svc2004/`. The data consist of genuine and forged signatures in English and Chinese, and the competitors' task was to distinguish the true signatures from the forgeries. Here our goal is different: focusing only on the genuine signatures, we use dbICC to assess whether the English and Chinese signatures differ in terms of test-retest reliability. The data consist of 20 replicates for each of 23 English and 17 Chinese signatures. Fig. W3 displays five of the 20 instances of Chinese and English signatures from two individuals.

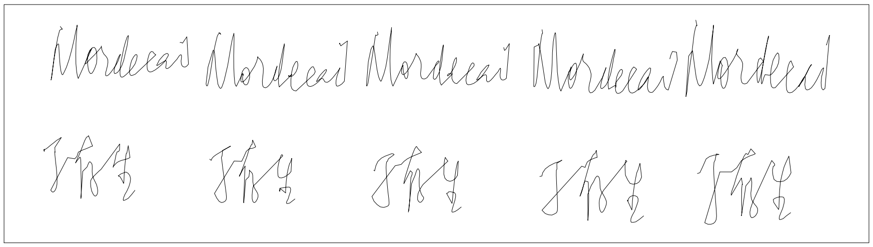

Figure W3: Examples of data from the Signature Verification Competition: five replicates of an English signature (above) and of a Chinese signature (below).

As in Reiss et al. (2017), we use dynamic time warping (DTW; Sakoe and Chiba, 1978; Giorgino, 2009) to define distance between each pair of signatures, yielding an 800×800 matrix of squared distances. In contrast to the setup of Section 5.2, DTW

distance is not induced by a norm, and is not even a metric; but this does not prevent us from applying the population version (3) and the estimate (6) of the dbICC. We find a dbICC of 0.84 for the English signatures, versus 0.71 for the Chinese signatures. A permutation test, based on 2000 random shuffles of the language labels for the 40 participants, provides only weak evidence ($p = 0.069$) of a difference in reliability.

References

Giorgino, T. (2009). Computing and visualizing dynamic time warping alignments in R: the dtw package. *Journal of Statistical Software 31*(7), 1–24.

Gower, J. C. (1966). Some distance properties of latent root and vector methods used in multivariate analysis. *Biometrika 53*(3-4), 325–338.

Jiang, T. (2019). Determinant of sample correlation matrix with application. *Annals of Applied Probability 29*(3), 1356–1397.

Kulis, B., M. A. Sustik, and I. S. Dhillon (2009). Low-rank kernel learning with Bregman matrix divergences. *Journal of Machine Learning Research 10*, 341–376.

Mardia, K. V., J. T. Kent, and J. M. Bibby (1979). *Multivariate Analysis.* New York: Academic Press.

Reiss, P. T., D. L. Miller, P.-S. Wu, and W.-Y. Hua (2017). Penalized nonparametric scalar-on-function regression via principal coordinates. *Journal of Computational and Graphical Statistics 26*(3), 569–578.

Sakoe, H. and S. Chiba (1978). Dynamic programming algorithm optimization for spoken word recognition. *IEEE Transactions on Acoustics, Speech and Signal Processing 26*(1), 43–49.

Shehzad, Z., C. Kelly, P. T. Reiss, R. C. Craddock, J. W. Emerson, K. McMahon, D. A. Copland, F. X. Castellanos, and M. P. Milham (2014). A multivariate distance-based analytic framework for connectome-wide association studies. *NeuroImage 93*, 74–94.

Walther, A., H. Nili, N. Ejaz, A. Alink, N. Kriegeskorte, and J. Diedrichsen (2016). Reliability of dissimilarity measures for multi-voxel pattern analysis. *NeuroImage 137*, 188–200.

Wood, S. N., N. Pya, and B. Säfken (2016). Smoothing parameter and model selection for general smooth models (with discussion). *Journal of the American Statistical Association 111*, 1548–1575.
