# Supplementary material for: Generalized reliability based on distances
Source: Biometrics. 2020 May 8;77(1):258–70. doi: 10.1111/biom.13287 (PMC7984087; doi:10.1111/biom.13287)
Supplement: Supplementary file 2 [file BIOM-77-258-s001.zip › README.html]

A brief guide to the <strong><code>dbicc</code></strong> package


# A brief guide to the **`dbicc`** package

This package implements a new approach to reliability, extending the classical intraclass correlation coefficient (ICC) to a new measure based on arbitrary distances among observations (Xu et al., 2020).

The key function `dm2icc` computes the reliability (distance-based ICC, or dbICC) for a given matrix of distances among observations, while `dm2icc.bt` gives boostrap confidence intervals for the dbICC value.

The dataset `dt2v3` is the fMRI data set analyzed by Xu et al. (2020). `plotdmat` visualizes the associated distance matrix.

Please feel free to contact Meng Xu mxu@campus.haifa.ac.il with questions and bug reports.

## Installation

This package can be installed from GitHub, and loaded, as follows:

```
# devtools::install_github("wtagr/dbicc", force = TRUE)
library(dbicc)
```

## Distance matrix computation

The package does not include functions to compute the distance matrix, but the following function performs this step for our application. It inputs a *list* object and outputs a distance matrix determined by the *method* argument: `method = 'f'` yields the Frobenius norm, `method = 'r'` the \(\sqrt{1-r}\) distance, and `method = 'l1'` the \(\ell\_1\) distance (sum of absolute differences).

```
mdist <- function(datalist, method=c("f")) {
    dmat<-matrix(0,length(datalist),length(datalist))
    for (i in 2:length(datalist)) for (j in 1:(i-1)){
        if (method=="r"){
            v1<-datalist[[i]][lower.tri(datalist[[i]], diag = FALSE)]
            v2<-datalist[[j]][lower.tri(datalist[[j]], diag = FALSE)]
            r<-cor(v1,v2)
            dmat[i,j]=dmat[j,i]=sqrt(1-r)
        } else if (method=='l1'){
            dmat[i,j]=dmat[j,i]=sum(abs(datalist[[i]]-datalist[[j]]))
        } else {
            dmat[i,j]=dmat[j,i]=norm(datalist[[i]]-datalist[[j]],type=method)
        }
    }
    return(dmat)
}
```

`dt2v3` contains fMRI time series for 25 individuals with 2 scans each, grouped by individual. Here we compute a matrix of distances among the correlation matrices resulting from these multivariate time series:

```
data(dt2v3)

# compute correlation matrices
cor2v3 <- lapply(dt2v3,cor)

# compute the matrix of distances among the correlation matrices
distmat <- mdist(cor2v3)

# visualize
plotdmat(distmat, 25, 2)
```

## Compute the reliability

We can then input the distance matrix to `dm2icc`, to compute the dbICC:

```
dm2icc(distmat,25,2)
```

```
## [1] 0.3783276
```

Obtain confidence interval for the dbICC, based on 100 boostrap replicates (a larger number of replicates is recommended in practice):

```
dm2icc.bt(distmat,25,2, nB=100)
```

```
##      2.5%     97.5% 
## 0.3300415 0.4273384
```

## Reference

Xu, M., Reiss, P. T., and Cribben, I. (2020). Generalized reliability based on distances. *Biometrics*, to appear.
